# Genetic vaccine for tuberculosis (pVAXhsp65) primes neonate mice for a strong immune response at the adult stage

**DOI:** 10.1186/1479-0556-5-12

**Published:** 2007-11-29

**Authors:** Ana Cláudia Pelizon, Douglas R Martins, Sofia FG Zorzella, Ana Paula F Trombone, Júlio CC Lorenzi, Robson F Carvalho, Izaíra T Brandão, Arlete AM Coelho-Castelo, Célio L Silva, Alexandrina Sartori

**Affiliations:** 1 Department of Microbiology and Immunology, Biosciences Institute, São Paulo State University (UNESP), Botucatu, São Paulo, 18618-000, Brazil; 2 Department of Morphology, Biosciences Institute, São Paulo State University (UNESP), Botucatu, São Paulo, 18618-000, Brazil; 3 Department of Biochemistry and Immunology, University of São Paulo (USP), Ribeirão Preto, São Paulo, 14049-900, Brazil

## Abstract

**Background:**

Vaccination of neonates is generally difficult due to the immaturity of the immune system and consequent higher susceptibility to tolerance induction. Genetic immunization has been described as an alternative to trigger a stronger immune response in neonates, including significant Th1 polarization. In this investigation we analysed the potential use of a genetic vaccine containing the heat shock protein (hsp65) from *Mycobacterium leprae *(pVAXhsp65) against tuberculosis (TB) in neonate mice. Aspects as antigen production, genomic integration and immunogenicity were evaluated.

**Methods:**

Hsp65 message and genomic integration were evaluated by RT-PCR and Southern blot, respectively. Immunogenicity of pVAXhsp65 alone or combined with BCG was analysed by specific induction of antibodies and cytokines, both quantified by ELISA.

**Results:**

This DNA vaccine was transcribed by muscular cells of neonate mice without integration into the cellular genome. Even though this vaccine was not strongly immunogenic when entirely administered (three doses) during early animal's life, it was not tolerogenic. In addition, pVAXhsp65 and BCG were equally able to prime newborn mice for a strong and mixed immune response (Th1 + Th2) to pVAXhsp65 boosters administered later, at the adult life.

**Conclusion:**

These results suggest that pVAXhsp65 can be safely used as a priming stimulus in neonate animals in prime-boost similar strategies to control TB. However, priming with BCG or pVAXhsp65, directed the ensuing immune response triggered by an heterologous or homologous booster, to a mixed Th1/Th2 pattern of response. Measures as introduction of IL-12 or GM-CSF genes in the vaccine construct or even IL-4 neutralization, are probably required to increase the priming towards Th1 polarization to ensure control of tuberculosis infection.

## 1. Background

Tuberculosis (TB) is a disease caused by *Mycobacterium tuberculosis *(*M. tuberculosis*) which affects mainly the lungs. It is a major public-health problem, with around 9 million new cases and 2 million deaths estimated to occur each year [1].

The attenuated BCG strain of *Mycobacterium bovis *has been extensively used as a vaccine against TB for the past several decades. The vaccine has many virtues, including the fact that it can be safely given to young children and is inexpensive to be produced. However, in spite of its wide use, a large number of well documented trials have shown that the protective efficacy of BCG may vary greatly, from 0 to 80% [2]. This highly variable and poorly protective efficacy in certain countries has been attributed to the various BCG strains used as vaccines, environmental factors and host genetic characteristics [3,4]. Although the overall efficacy is low, one important observation that is shared by most studies, is that BCG vaccine protects against disseminated disease in newborns and children. In addition, this immunity wanes with age, resulting in insufficient protection against adult pulmonary TB [5,6]. Besides protection against more severe forms of TB in young children, recent reports have strongly reinforced the role of bacillus Calmette-Guérin as an immunomodulator for prevention and treatment of allergy, asthma and autoimmune diseases [7,8].

In this context, there is a great interest in the development of new vaccines against TB. A number of alternative living and non-living putative TB vaccines are being studied and discussed by many authors [9-11]. DNA vaccines have been successful in several experimental infection models and some reports provide evidence of their feasibility for TB control. DNA constructs encoding mycobacterial antigens as 65-kDa heat shock protein (hsp65), Ag85A, Ag85B and ESAT-6 induced significant protective immunity [12-14]. Additionally, attempts to improve BCG by administering lower doses, oral delivery and prime-boost protocols are being explored [15,16]. An heterologous prime-boost regimen, which boosts or augments BCG or rBCG, is being considered the most realistic strategy for future TB control through immunization [6].

As a new TB vaccine will be administered to human neonates, it must be realized that newborns and young infants from numerous animal species show limitations in generating protective immune responses. Neonatal murine immunization models using conventional vaccine antigens (measles, tetanus toxoid) in BALB/c mice gave responses similar to those found early in human infants. Early life B cell responses generally resulted in a slower and weaker increase of vaccine antibodies compared with adult mice. Furthermore, analyses of T cell responses to these conventional vaccines indicated that early life T cell differentiation was preferentially polarized towards a Th2 pattern [17]. As a consequence of this Th2 bias, there is a deficient production of IFN-γ, TNF-α and CTL responses, considered essential for protection against many intracellular pathogens.

In this investigation we analysed the potential use of a genetic vaccine (pVAXhsp65) against TB in neonate mice. Aspects as presence of hsp65 message in different tissues, genome integration and immunogenicity in homologous and heterologous prime-boost strategies were evaluated.

## 2. Materials and methods

### 2.1 Mice

BALB/c mice were bred in the Animal Facility of São Paulo State University (UNESP) at the Biosciences Institute and used at 5 (neonates), 12, 19 and 30-day-old. Breeding cages were checked daily for new births and the day of birth was recorded as the day the litter was found. Pups were kept with the mothers until they were weaned at 21-day-old. The animal protocols used in this work were approved by the local ethical committee that follows the guidelines adopted by the Brazilian College of Animal Experimentation (COBEA).

### 2.2 Plasmid DNA construction and purification

The vaccine pVAX-hsp65 was derived from the pVAX vector that use the CMV intron (Invitrogen^®^, Carlsbad, CA, USA), previously digested with BamH I and Not I (Gibco BRL, Gaithersburg, MD, USA) to insert a 3.3 kb fragment corresponding to the *M. leprae *hsp65 gene. The empty pVAX vector was used as a control. DH5α *E. coli *transformed with plasmid pVAX or the plasmid carrying the hsp65 gene (pVAX-hsp65) were cultured in LB liquid medium (Gibco BRL, Gaithersburg, MD, USA) containing kanamicin (50 μg/ml). The plasmids were purified using the Concert High Purity Maxiprep System (Gibco BRL, Gaithersburg, MD, USA). Plasmid concentrations were determined by spectrophotometry at λ = 260 and 280 nm by using the Gene Quant II apparatus (Pharmacia Biotech, Buckinghamshire, UK).

### 2.3 Vaccines and immunization procedures

In addition to the genetic vaccine (pVAXhsp65) described above, we also used BCG Moreau strain. Young mice including newborns were immunized with 50 μg of pVAXhsp65 (20 μl) in the quadriceps muscle, whereas adults were immunized with 100 μg of pVAXhsp65 (100 μl). For adults, but not young mice, 10% of saccharose was added. Corresponding control groups received saline or pVAX in the same conditions. In the prime-boost type of experiments, mice received one dose of pAXhsp65 (50 μg) or BCG (10^5^CFU) at 5-day-old and then, at the adult life, were boostered with two pVAXhsp65 doses (100 μg each) administered 30 and 45 days after, respectively.

### 2.4 Isolation of RNA and detection of hsp65 mRNA by RT-PCR

At various periods of time (48 and 72 hours and 7 days) after the administration of pVAXhsp65, samples from muscle, draining lymph nodes, spleen, thymus, liver, kidney, lung and heart were obtained. The samples were treated with Trizol reagent (Invitrogen, Carlsbad, CA, USA) and total RNA was isolated according to the manufacturer protocol. Subsequently, RNA was extracted with chloroform and precipitated with isopropyl alcohol. The total extracted RNA was dissolved in nuclease-free water (Invitrogen). Total cellular RNA (10 ug) was reversed transcribed using oligo (dT) primers and reverse transcriptase (Invitrogen) following manufacturer instructions. The contaminating plasmid DNA was previously removed by treatment with DNAse I, amplification-grade (Invitrogen). The cDNA (2 ug) was amplified for 35 cycles at 94°C for 30 seconds, 60°C for 45 seconds and 72°C for 1.5 minutes, using the primer pair 5'-ACC AAC GAT GGCGTG TCC AT-3' and 5'-TAG AAG GCA CAG TCG AGG-3', resulting in a 400-bp cDNA encoding hsp65, or the primer pair 5'-GTG GGC CGC TCT AGG CAC CAA-3' and 5'-CTC TTT GAT GTC ACG CAC GAT TTC-3', resulting in a 450-bp cDNA encoding β-actin.

### 2.5 Quantification of anti-hsp65 antibodies

Serum samples were collected by retro-orbital bleeding two weeks after the last DNA dose and anti-hsp65 specific antibody levels were evaluated by enzyme-linked immunosorbent assay (ELISA). Maxisorp plates (Nunc) were coated with 0,1 ml of purified recombinant hsp65 (5 μg/ml) in coating solution (14.3 mM Na_2_CO_3_, 10.3 mM NaHCO_3_, pH 9.6), incubated at 4°C overnight and then blocked with 10% fetal calf serum (FCS) in PBS for 60 minutes at 37°C. Serum samples diluted 1:25 were tested. After incubation for 2 hours at 37°C, anti-mouse IgG1 and IgG2a biotinylated conjugates (A85-1 and R19-15, respectively, from PharMingen), were added for detection of specific isotype antibodies. After washing, plates were incubated at room temperature for 30 minutes with StreptAB kit (Dako, Carpinteria, CA, USA) and then revealed by adding H_2_O_2 _+ OPD. Color development was stopped with H_2_SO_4 _and optical density was measured at 492 nm.

### 2.6 Evaluation of cytokine production

Two weeks after the last DNA dose the animals were sacrificed and splenic cells were collected and adjusted to 5 × 10^6 ^cells/ml in RPMI 1640 medium, supplemented with 5% FCS, 20 mM glutamine and 40 IU/l of gentamicin. The cells were cultured in 48-well flat-bottomed culture plates (Nunc, Life Tech. Inc., Maryland, MA, US) in the presence of 40 μg/ml of Concanavalin A (ConA). Cytokine levels in culture supernatants were evaluated 48 hours later by ELISA. Cytokines were measured following manufacturer instructions (PharMingen). Purified monoclonal antibodies anti-IFN-γ (R4-6A2), IL-4 (11B11) and IL-5 (TRKF5) were used at 1 μg/ml as capture antibodies and the following biotinylated antibodies were used for detection: anti-IFN-γ (XMG1.2); IL-4 (BVD6) and IL-5 (TRFK4) at 0,5 μg/ml.

### 2.7 Statistical analysis

Results are expressed as the mean +/- SEM for each variable. Statistical analysis was performed using Minitab Version 1996 (Minitab Inc, State College, PA, USA). One-way ANOVA and the Fisher test were used to compare cytokine and antibody levels. Values of p < 0,05 were considered statistically significant.

## 3. Results

### 3.1 pVAXhsp65 is transcribed in neonate mice immunized by intramuscular route

**Figure 1:**
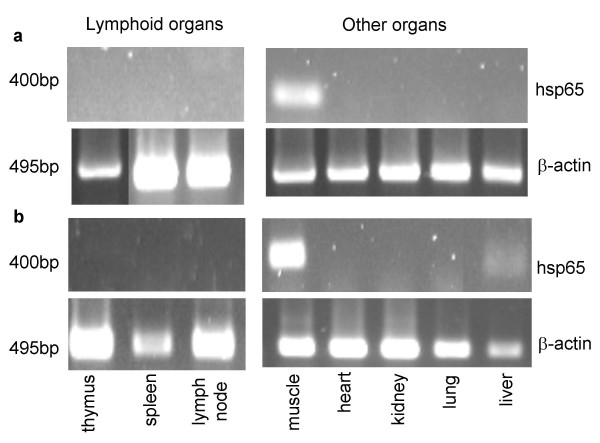
**Tissue distribution of hsp65 message**. The presence of hsp65 message was evaluated in different tissue samples collected 48 hours (a) and 7 days (b) after intramuscular injection of 50 ug of pVAXhsp65. Total RNA (10 ug) isolated from each tissue was treated with DNase and subjected to RT-PCR amplification with specific primers. β-actin was amplified as an RNA quality control. All RT-PCR products were analysed by agarose gel electrophoresis and visualized by ethidium bromide staining. Similar results were observed in two animals analysed for each period. No products were seen (hsp65 and β-actin) when total RNA was subjected to PCR amplification in the absence of a previous reverse transcription.

The presence of mRNA for hsp65 was evaluated by RT-PCR in various tissues at different time points (48 and 72 hours and 7 days) after intramuscular pVAXhsp65 immunization. Fourty-eight hours after immunization, hsp65 transcripts were found in the quadriceps muscle (vaccination site) but not in any of the other tissues examined as thymus, spleen, popliteal lymph nodes, liver, lung, heart and kidney. On days 3 and 7, hsp65 message was still present in the muscle but did not appear in any of the other organs. Hsp65 message also appeared in the liver of one animal (from three tested) at day 7. As expected, hsp65 transcripts were not detected in tissues from animals injected with the empty plasmid DNA vector (data not shown). Also, message for β-actin was detected in all evaluated samples demonstrating the suitability of RNA samples for this analysis. The results observed at 48 hours and 7 days are shown in Figure 1a for lymphoid organs and 1b for the other organs.

### 3.2 pVAXhsp65 has immunomodulatory properties in young mice

**Figure 2:**
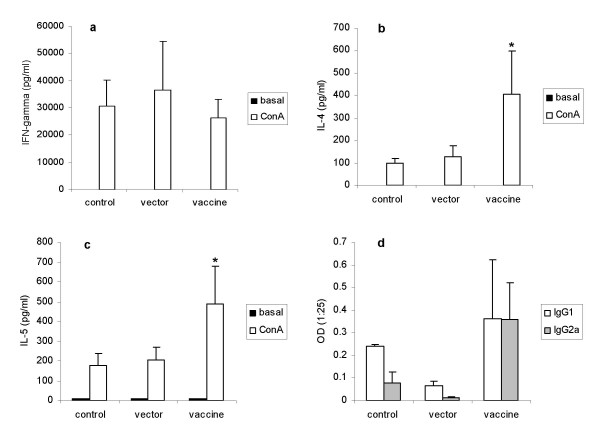
**Immunomodulatory activity of pVAXhsp65 in young mice**. Young mice received 3 pVAXhsp65 doses (50 μg/im route) delivered at 5, 12 and 19-day-old. Production of IFN-γ (a); IL-4 (b) and IL-5 (c) by splenic cells stimulated with ConA and serum levels of specific anti-hsp65 antibodies (d) were determined two weeks later. Results represent the geometric mean ± SEM of 4 to 8 individually tested animals per group. *p < 0.05 in comparison to vector group.

Young mice received three pVAXhsp65 intramuscular doses delivered at 5, 12 and 19 days of age. Fifteen days after the last dose they were sacrificed and Th1/Th2 profile were tested by both, splenic cytokine production in response to ConA stimulation and anti-hsp65 IgG1 and IgG2a serum levels. The most prominent alteration was a significantly higher production of Th2 cytokines (IL-4 and IL-5, shown in Figures 2b and 2c, respectively) in mice that received pVAXhsp65 in comparison to the control ones (not injected or injected with the empty vector). A discrete and variable increase (with no statistical significance) in the production of both isotypes, IgG1 and IgG2a anti-hsp65, was detected and can be observed at Figure 2d. pVAXhsp65 did not affect IFN-γ production in a significant way (Fig. 2a).

### 3.3 pVAXhsp65 is not tolerogenic for young mice

**Figure 3:**
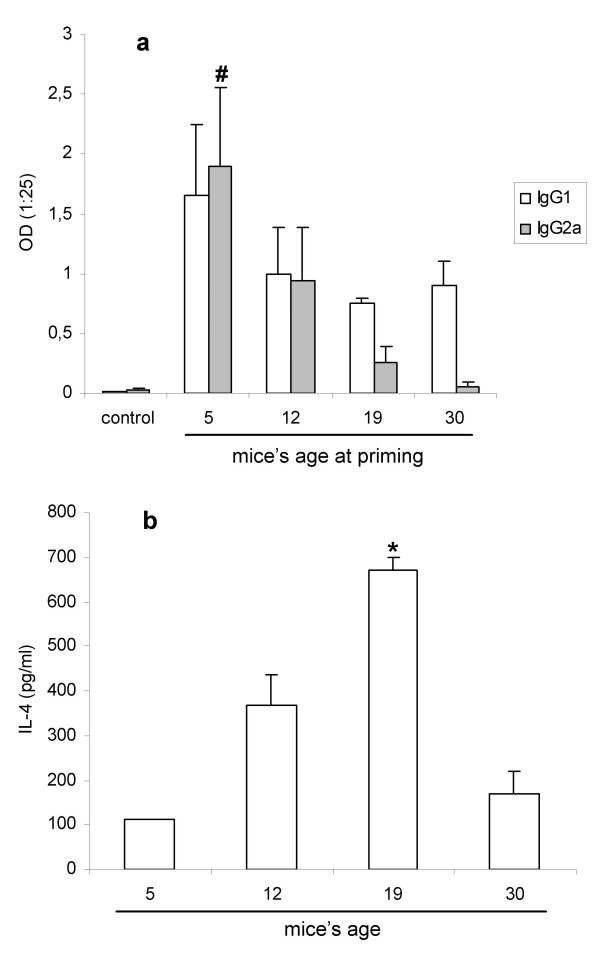
**Effect of mice's age on priming by pVAXhsp65 (a) and on IL-4 production (b)**. BALB/c mice were primed with pVAXhsp65 at distinct ages (5, 12, 19 and 30 days) and boostered with this vaccine 4 weeks later. Antibody serum levels were evaluated by ELISA 2 weeks after the booster. Results represent the geometric mean ± SEM of 4 to 8 animals individually tested per group. Ability to produce IL-4 was tested in supernatants from splenic cells in mice with 5, 12, 19 and 30-day-old stimulated *in vitro *with ConA. Results represent the geometric mean ± SEM of 5 animals individually tested, except for the 5 days old group that was tested by a pool of cells. # p < 0.05 in comparison to 19 and 30 days; * p < 0.05 in comparison to the other groups.

To evaluate the tolerogenicity of pVAXhsp65 for young BALB/c mice, animals received a first DNA dose at distinct ages (5, 12, 19 or 30-day-old) and were then challenged, three weeks later, at the adult life, with another DNA dose. Fifteen days after last DNA dose, the serum levels of IgG1 and IgG2a anti-hsp65 antibodies were determined. The results shown in Figure 3a demonstrated that this vaccine was not tolerogenic because high specific antibody levels were detected after the dose administered at the adult stage. A clear effect of the age on the preferential priming for IgG2a was observed; the earlier the vaccine was injected, the higher was the specific IgG2a level (Fig. 3a). IgG2a anti-hsp65 antibodies were significantly higher in mice whose priming occurred at 5-day-old, in comparison to 19 or 30-day-old. Specific IgG1 levels were not affected by the age of the animal during priming. Interestingly, the ability to produce IL-4 in response to polyclonal stimulation with Con A was much higher in animals with 12 and 19 days, in comparison to 5-day-old (Fig. 3b).

### 3.4 pVAXhsp65 and BCG similarly prime neonate mice for a strong and mixed (Th1/Th2) anti-hsp65 response at the adult stage

**Figure 4:**
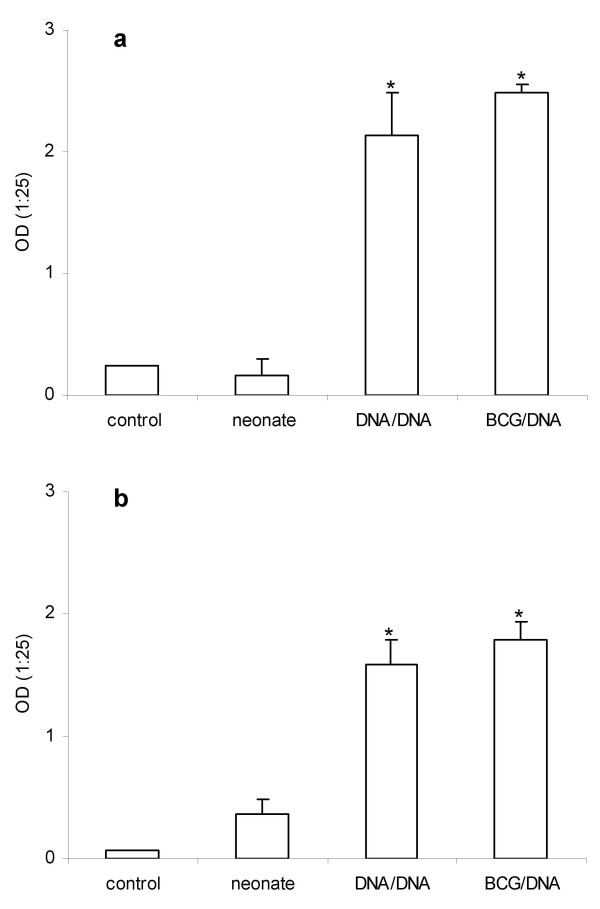
**Comparative priming of neonate mice with pVAXhsp65 and BCG for specific anti-hsp65 antibody production**. Five-day-old mice received a priming dose of pVAXhsp65 or BCG and then two pVAXhsp65 doses at the adult phase; experimental groups were identified as DNA/DNA and BCG/DNA respectively. A non-immunized group and a group immunized with 3 pVAXhsp65 doses delivered at 5, 12 and 19-day-old were identified as control and neonate, respectively. Two weeks after last dose, the serum levels of IgG1 (a) and IgG2a (b) anti-hsp65 antibodies were evaluated by ELISA. Results represent the geometric mean ± SEM of 6 – 8 individually tested animals per group. *p < 0.05 in comparison to neonate group.

Neonate mice (5-day-old) were primed with one dose of pVAXhsp65 or BCG and boostered 4 weeks later with two doses of pVAXhsp65, delivered two weeks apart. As can be observed in Figure 4, both strategies triggered a significant increase in the levels of IgG1 and IgG2a anti-hsp65 antibodies, shown at Figure 4a and 4b, respectively. The priming effect of BCG and pVAXhsp65 seemed to be very similar in intensity and quality because no statistical differences were observed in specific IgG1 and IgG2a antibodies when these two protocols were compared.

## 4. Discussion

This report provides evidence that pVAXhsp65, a genetic vaccine constructed by insertion of the *M. leprae *hsp65 gene into a plasmid bacterial vector (pVAX), is transcribed by muscular cells of neonate mice. Additionally it shows that even though it was weakly immunogenic when entirely (3 doses) delivered during neonatal life, one dose of this vaccine was able to strongly prime the immune system of neonate mice to respond to a booster administered later during the adult stage. We also observed that BCG was able to prime neonate mice to respond to pVAXhsp65 later, in the adult life.

This investigation was initiated by evaluation of mRNA for hsp65, by RT-PCR, in different tissues. Using only one dose of 50 μg of pVAXhsp65 by intramuscular delivery, we observed that hsp65 message was always present in the muscle, i.e., at the inoculation site, even 7 days post-immunization. This message was not found in secondary lymphoid organs as spleen and lymph nodes as would be expected from our previous results employing a very similar vaccine in adult BALB/c mice [18]. The absence of mRNA for hsp65 in secondary lymphoid organs could explain, at least partially, the very low humoral immune response induced by three doses of DNA delivered during early life. However, this transcription limited to the inoculation site seemed sufficient to prime the animals for a strong immune response induced later, at the adult life. Absence of transcripts in the thymus was considered important because the presence of mRNAhsp65 in the thymus could be a concern, since the expression of antigen in this tissue could induce tolerance by deletion of hsp65-specific clones, altering the induction of specific immunity after an immunization schedule [19].

Although we have shown before that a similar tuberculosis vaccine did not integrate into the host cellular genome [18], this kind of evaluation was considered mandatory in very young mice (5-day-old). Due to their inherent high cellular proliferative activity they could be more prone to present plasmid integration into the genome. In addition, this new vaccine construction (pVAXhsp65), that was not previously tested and that could be acceptable for future human studies, was adopted in this investigation. Also, even though mRNA for hsp65 was found only in muscle tissue, we could not exclude the presence of plasmid DNA in other organs as spleen, liver, thymus and regional lymph nodes. Therefore, Southern blot analysis was conducted at different time points after immunization (48 and 72 hours and 7 days) in these tissues and also in muscle samples. This analysis demonstrated that pVAXhsp65 did not integrate into the DNA at any time analyzed (data not shown), confirming our previous observations in adult BALB/c mice [18]. Although higher sensitive methods are necessary to definitively exclude a possible genomic integration, these results are promising in the context of new procedures for neonatal immunization with DNA vaccines. These results are also in accordance with previous reports that showed a negligible risk of genomic integration after intramuscular inoculation of different plasmid constructions [20-22].

Like many other vaccines designed for human use, it must be considered that a new TB vaccine will be administered to newborn children. With this in mind we evaluated pVAXhsp65 immunogenicity for young mice. The immunization schedule included 3 DNA doses delivered during neonatal stage. The discrete immune response induced by three consecutive DNA doses, all delivered before weaning, could mean that the protocol (3 doses in 14 days) was not highly immunogenic. The very short interval between vaccine doses could be responsible for the observed low immunogenicity. This possibility is clearly supported by the work of Leitner *et al.*[23]. These authors demonstrated that expanding the interval between the doses of a DNA vaccine for malaria (circumsporozoite protein from *Plasmodium berghey*) gave the strongest effect, increasing efficacy and antibody boosting. However, a state of partial tolerance could be also induced, due not only to the high frequency of vaccination exposure but also to the fact that neonate mice are much more prone to develop tolerance depending on the conditions of antigen exposure [24]. To more directly test the ability of this construction to induce tolerance, we used a more appropriate and classical protocol that included one vaccine dose in neonates followed by a second dose during adult life. In this case, high levels of specific IgG2a and IgG1 were induced, demonstrating that one pVAXhsp65 dose delivered during neonatal stage was not tolerogenic. This absence of neonatal tolerance was different from results obtained with a genetic vaccine for malaria [25] but was in accordance with other investigations that showed no tolerance induction by genetic vaccines in neonates [26]. Interestingly, this priming effect was strongly influenced by the age of the animal; the highest IgG2a levels, what suggest Th1 stimulation, were observed in animals primed at 5-day-old. These findings could be explained, at least partially, by the differential production of IL-4 at these periods; highest IL-4 levels coincided with the lowest IgG2a production during the young stage.

Two main reasons make us to believe that regulatory T cells (Treg cells) could be also involved in this low immune response to pVAXhsp65 vaccination in neonates. Even though they are not fully characterized, there are enough experimental evidences showing that they can be natural or induced and that they control immune response to pathogens, tumors and even to self components [27-29]. The first indication that Treg cells could be important in the neonatal context came from experiments showing that neonatal thymectomy lead to an increased incidence of autoimmune diseases [30,31]. More recently, the contribution of natural Treg cells during pregnancy, maintaining maternal tolerance to the fetus, was described [32]. A high proportion of CD4+CD25+ natural Treg cells was also demonstrated in cord blood, being particularly higher in premature babies compared to full-term babies [33]. Interestingly, these cells express Treg cell markers as CTLA-4 and Foxp3 and also exert potent immunosuppressive activity over proliferation and cytokine production following stimulation with specific antigen [33,34]. Besides this, the gene inserted in this genetic construction codes for the heat shock protein (hsp65) from *Mycobacterium leprae*. The contribution of hsps (mainly hsp60/65) to control inflammation associated with autoimmune diseases has been abundantly reported, including by researchers from our group [35-37]. Hsp peptides and plasmid vaccines constructed with hsp genes have been even tested in clinical trials due to their ability to activate hsp-specific T reg cells [38]. In this context, we could hypothesize that vaccination with pVAXhsp65 during neonatal life is activating both, natural and induced Treg cells, triggering therefore, an excessive and early regulation of the immune response.

Even though the humoral specific immune response was discrete, this immunization schedule was associated with a strong immunomodulatory effect over the immune response of vaccinated mice, characterized by higher IL-4 and IL-5 levels found in splenic cell cultures stimulated with ConA. The origin of the elevated production of IL-4 and IL-5 was not investigated. However, based on the characteristics of the neonate immune response, we could imagine that a combination of different factors contributed to this immunomodulation. First, we believe that this effect is related to the heat shock protein itself or its encoding gene because only vaccinated animals (and not the vector injected ones) presented this modulation. The most simple explanation could be a strongly skewed Th2 response associated with a possible high amount of antigen produced in a short period of time. Literature reports on neonatal immunity support this possibility. Mouse peripheral T cells in the first few weeks of life are a mixture of fetal and adult derived cells and these CD4^+ ^T cells of fetal origin mount a Th2 skewed response in an antigen-dose-dependent manner [39,40]. Interestingly, even genetic vaccines that are claimed to be stronger Th1 inducers in adult animals triggered Th1/Th2 mixed responses in neonates [41,42]. This neonatal Th2 bias has been even envisaged as a valuable tool for prophylaxis of autoimmune diseases [43]. It is important to highlight, however, that this elevated production of Th2 cytokines could jeopardize resistance to intracellular pathogens by, for example, decreasing Th1 expression as has been discussed in the context of new tuberculosis vaccines [44].

As priming with pVAXhsp65 in 5-day-old mice triggered a strong priming for IgG2a, we compared this with a BCG priming delivered at the same period, i.e. 5-day-old. Both groups were boostered with two pVAXhsp65 doses in the adult phase. pVAXhsp65 and BCG similarly primed for a strong humoral response, characterized by high levels of both, IgG1 and IgG2a. These results suggest that both, the genetic pVAXhsp65 vaccine and BCG were able to prime neonate mice for a strong immune response to pVAXhsp65 boosters delivered later, in the adult life. Even though these strategies appear promising in the search for a new TB vaccine, the mixed Th1/Th2 response will not, probably, be able to control TB infection. This assumption is based on the extensive literature that considers these aspects [45-47].

## 5. Conclusion

Together, the observed results suggest that this genetic vaccine is safe and very powerful to prime neonate mice immune system. This could be further explored with protocols designed to shape the induced immunity to a protective kind of response against TB. An attractive possibility is to reinforce Th1 polarization during neonatal period by addition of GM-CSF and IL-12 plasmids or even CpG motifs.

## 6. Authors' contributions

ACP and AS are the principal investigators in this study. DRM and SFGZ largely contributed with the immunological experiments. APFT and RFC helped with RT-PCR. JCCL and AAMCC carried out the southern blot. ITB was responsible for production of vaccine and rhsp65. CLS provided critical input and assistance.
